# Feasibility of Large Language Model–Based Standardized Virtual Patients to Support Clinical Decision-Making Training in Operative Dentistry: Mixed Methods Study

**DOI:** 10.2196/91021

**Published:** 2026-05-19

**Authors:** Fahad BaHammam

**Affiliations:** 1 College of Dentistry, King Saud bin Abdulaziz University for Health Sciences, Prince Mutib Ibn Abdullah Ibn Abdulaziz Rd, Ar Rimayah, Riyadh, Saudi Arabia, 966 114295941; 2 King Abdullah International Medical Research Center, Riyadh, Saudi Arabia; 3 Dental Services, Ministry of National Guard - Health Affairs, Riyadh, Saudi Arabia

**Keywords:** large language models, clinical decision-making, dentistry, proof-of-concept study, feasibility studies, education, simulation training, standardized patients, virtual patients, operative, dental

## Abstract

**Background:**

Clinical decision-making training in operative dentistry commonly relies on real or standardized patients to develop undergraduate students’ ability to deliver safe, effective, and patient-centered care. However, training with real or standardized patients can be limited in scalability, cost-effectiveness, and accessibility. Large language models, with their human-like language capabilities, may have the potential to simulate patients in clinical encounters and help overcome some limitations associated with traditional training approaches.

**Objective:**

This study aimed to evaluate the feasibility of using large language model–based standardized virtual patients to support undergraduate dental students’ clinical decision-making training in operative dentistry.

**Methods:**

This mixed methods cross-sectional feasibility study was conducted during a simulation-based clinical decision-making training session in the Operative Dentistry and Cariology course at the College of Dentistry, King Saud bin Abdulaziz University for Health Sciences, Riyadh, Saudi Arabia. Eligible participants were second-year undergraduate dental students enrolled in the course. A convenience sampling approach was used, with all eligible students (N=50) invited to participate. A total of 41 students completed the study, 23 (56%) of whom were male. The students were divided into 8 groups. Each group interacted with 2 standardized virtual patients powered by ChatGPT-4o (OpenAI) through the Chatbase platform to complete comprehensive history-taking and then reviewed the standardized virtual patients’ intraoral photographs and bitewing radiographs. For each standardized virtual patient, students as a group recorded diagnoses, performed a risk assessment, and formulated a treatment plan. Students then completed the Student Satisfaction and Self-Confidence in Learning questionnaire. The quality of the standardized virtual patient responses and overall dialogue realism were evaluated using the Dialogue Authenticity Scale. The dialogues were also thematically analyzed to identify authenticity-undermining response features and explore their context and underlying causes.

**Results:**

Students perceived the simulation-based training session positively, with all questionnaire items showing high median scores (4.00‐5.00 on a 5-point scale), and both item-level IQRs and 95% CIs spanning no more than 1.0 scale point. In addition, standardized virtual patient responses were largely authentic, with an overall median authenticity rating of 4.50 (IQR 4.00-5.00; 95% CI 4.00-5.00) on a 6-point scale across all interactions. However, several authenticity-undermining response features were identified, including responses that were inconsistent with typical human behavior, contained information beyond a patient’s likely knowledge, or were factually incorrect.

**Conclusions:**

This proof-of-concept study supports the feasibility of implementing large language model–based standardized virtual patients in undergraduate simulation-based clinical decision-making training in operative dentistry. In a dental context where this application has been only minimally evaluated, this study provides early evidence of positive student perceptions, acceptability, and largely authentic dialogue, while also identifying important performance limitations. Further research is warranted to optimize performance and to evaluate the educational effectiveness of this approach in improving undergraduate students’ clinical skills and knowledge.

## Introduction

Clinical decision-making in operative dentistry is an iterative process in which a dentist gathers, evaluates, and interprets relevant patient information to determine the most likely diagnosis and decide on the most appropriate treatment plan [1-3]. It also integrates the best available evidence with clinical expertise and the patient’s values and preferences [1,2]. Training undergraduate students in clinical decision-making in operative dentistry is crucial because it ensures safe, effective, and patient-centered care across diagnosis, risk assessment, and treatment planning [3].

Traditional clinical decision-making training usually involves interactive encounters with real patients or standardized patients (trained human actors portraying patient scenarios) [4]. Although both approaches can improve students’ clinical decision-making capabilities, they have drawbacks that may limit their effectiveness and broader implementation [5]. For instance, involving real patients offers limited standardization across learners due to the wide spectrum of signs and symptoms that patients can present with [6]. In addition, the availability of suitable patients can be unpredictable [7]. Furthermore, ethical concerns can be raised when exposing patients to inexperienced learners [7]. On the other hand, using standardized patients is resource-intensive and associated with high costs related to recruitment, payment, training, and coordination, which can reduce the availability of standardized patient simulation training for students [6,7]. Lastly, standardized patients may experience fatigue and emotional burden when portraying distressing scenarios repeatedly [7].

Large language models are an emerging artificial intelligence technology that can generate human-like language, which gives them the potential to simulate patients in clinical encounters [8]. Although a recent comprehensive review by Puleio et al [9] synthesized the rapidly expanding applications of one of the most widely used models (ChatGPT) across the clinical, research, and educational domains of dentistry, many specific applications within operative dentistry education, including the simulation of patients in clinical decision-making training, remain underexplored [10]. To the best of our knowledge, only one innovation report has described the use of a large language model–based standardized virtual patient to support clinical decision-making training in dentistry without comprehensive evaluation [11].

Within the wider health care education field, large language model–based standardized virtual patients have been evaluated across several educational applications, including history taking, counseling, difficult conversations, cultural dexterity training, and clinical reasoning, with promising results [12-19]. These standardized virtual patients have been shown to generate reasonably authentic responses and provide appropriate feedback for learners [15,20,21]. They have also been demonstrated to enhance scalability, cost-effectiveness, and accessibility [20,21]. In addition, incorporating these standardized virtual patients into simulation training has been associated with improvements in students’ clinical skills and knowledge [13,22]. For instance, a randomized controlled trial found that medical students who received additional training with standardized virtual patients performed significantly better on the Pre-Clinical Clerkship Objective Structured Clinical Examination than the comparison cohort [22]. Finally, students have reported perceiving these standardized virtual patients as efficient and versatile learning tools [23].

Several challenges and limitations, however, may limit the practical use of these large language model–based standardized virtual patients. For example, large language models are susceptible to hallucinations, which can be defined as responses that seem plausible and reasonable but are incorrect [24]. Another possible limitation is that large language models have been observed to occasionally deviate from prompt instructions [25]. Additionally, the process by which large language models generate responses is not transparent, which can raise concerns regarding trust, accountability, and ethical risks [26].

Given the limited evidence on this innovative approach in operative dentistry education, this study aimed to evaluate the feasibility of using large language model–based standardized virtual patients to support undergraduate dental students’ clinical decision-making training in operative dentistry. This aim was addressed through 2 objectives. First, this study evaluated the quality of standardized virtual patient responses during student interactions and the realism of the overall dialogue in the simulation-based training session using the Dialogue Authenticity Scale [20]. Second, it explored students’ perceptions and the acceptability of the simulation-based training session using the Student Satisfaction and Self-Confidence in Learning questionnaire [27].

## Methods

### Overview

This study employed 2 large language model–based standardized virtual patients powered by ChatGPT-4o (OpenAI) within a simulation-based training session to evaluate their feasibility in supporting undergraduate clinical decision-making training in operative dentistry. The 2 standardized virtual patients were designed as high-caries-risk cases with differing risk factors and distinct demeanors and communication styles. Feasibility was evaluated using the Dialogue Authenticity Scale to assess the quality of standardized virtual patient responses during interactions with students and the realism of the overall dialogue in the simulation-based training session [20]. In addition, students’ perceptions and acceptability after the simulation-based training session were assessed with the validated Student Satisfaction and Self-Confidence in Learning questionnaire [27].

### Setting and Participant Characteristics

This study was conducted among the second-year undergraduate dental students at the College of Dentistry, King Saud bin Abdulaziz University for Health Sciences (KSAU-HS), Riyadh, Saudi Arabia, who were enrolled in the Operative Dentistry and Cariology course (RSTO 412) during the academic year 2024 to 2025. The study took place in the preclinical simulation laboratory during a training session within this course that focused on clinical decision-making in operative dentistry, with an emphasis on dental caries management.

### Sample Size

As this was a feasibility study for which a formal power calculation was not required, a convenience sampling approach was adopted by inviting the entire cohort of second-year dental students enrolled in the RSTO 412 course (N=50) to participate. This sample size is comparable to previous feasibility studies evaluating large language model–based standardized virtual patients in other medical education fields (46‐56 participants) [18,22].

### Development and Initial Evaluation of Standardized Virtual Patients

Two detailed system prompts were developed to create 2 standardized virtual patients. Each prompt specified the patient’s age, gender, chief complaint, history of the complaint, medical history, dental history, and social history. The system prompts also described the patient’s behavior, attitude, and concerns and provided general instructions on how to behave and respond during interactions with students. The standardized virtual patients were built using Chatbase, a platform for developing custom artificial intelligence chatbots based on ChatGPT-4o. Using this platform enabled the deployment of the standardized virtual patients while keeping participating students blinded to the system prompts. Chatbase also provides built-in functionality for recording interactions, which allowed the dialogues to be recorded and subsequently analyzed after the completion of the study.

The first standardized virtual patient was designed as a young, healthy, cooperative, male patient with dental-related pain associated with a deep carious lesion and multiple risk factors for dental caries. The second standardized virtual patient was designed as an older, medically compromised female patient who is at high caries risk and requires dental management before head-and-neck radiotherapy. While the 2 standardized virtual patients were not based on specific real patients, they were supplemented by anonymized patient-derived intraoral photographs and bitewing radiographs.

The initial performance of the standardized virtual patients was evaluated by 3 independent KSAU-HS College of Dentistry faculty members who reviewed the system prompts and then interacted with the standardized virtual patients. Adjustments were subsequently made to the system prompts based on their feedback. For example, modifications were applied to the system prompts to prevent the standardized virtual patients from deviating from their designated roles, as in some instances, the standardized virtual patients began asking the faculty members how they could help them. In addition, more personalized emotional depth was specified to increase the realism of the interactions. Copies of the final versions of the 2 system prompts are available in Multimedia Appendix 1.

### Study Procedures and Data Collection

Participating students were divided into 8 groups of 5 to 6 students. Each group first interacted in English with the first standardized virtual patient through a text-based interface using a separate, newly initiated chat instance. No conversation history or persistent bot state was carried over between groups. The interaction was conducted to complete a comprehensive history-taking exercise. All interactions between students and the standardized virtual patient were recorded and stored in the Chatbase cloud service. Each group documented their key findings from the history-taking exercise in the clinical case record form, including the patient’s chief complaint, as well as dental and medical histories. Each group then reviewed and examined the patient’s intraoral photographic images and bitewing radiographs and recorded diagnoses of dental caries based on their interpretation of the photographs and radiographs. They also performed a caries risk assessment based on the findings from the history-taking and dental caries diagnoses, using the American Dental Association Caries Risk Assessment Tool [28]. Lastly, they developed a comprehensive operative dentistry treatment plan for the patient that followed the International Caries Classification and Management System guidelines [29]. Once completed, an instructor discussed the findings and the treatment plan with the students and provided feedback.

A similar process, from history-taking to instructor feedback, was then conducted with the second standardized virtual patient. The only difference was that the caries risk assessment and the comprehensive treatment plan were based on the Caries Management by Risk Assessment (CAMBRA) guidelines [30]. Finally, students completed the validated Student Satisfaction and Self-Confidence in Learning questionnaire [27], which was administered online through Microsoft Forms (Microsoft Corporation).

### Statistics and Data Analysis

The responses of the standardized virtual patients during their 16 interactions with students, along with the overall dialogue, were rated at the interaction level (one rating per interaction) using the validated Dialogue Authenticity Scale by the study’s sole author [20], in accordance with the instrument’s scoring rubric. This instrument evaluates the quality and authenticity of dialogues with large language model–based standardized virtual patients across 5 main items, as shown in Table 1. Each item is evaluated using a 6-point Likert scale ranging from “strongly disagree” to “strongly agree,” where a higher score indicates stronger agreement. The 16 interactions were rated twice, with a 2-week washout period between rounds. Quadratic-weighted Cohen *κ* was calculated to assess intrarater reliability between Round 1 and Round 2, which yielded a quadratic-weighted kappa (*κ*_w_) value of 0.42, indicating moderate intrarater agreement. Areas of disagreement were reviewed against the rubric and documented, and a final rating was assigned with a documented justification. Then, all 16 interactions were rereviewed to ensure that the decisions made to resolve disagreements were consistently applied. The ratings of the 16 interactions were summarized using medians and IQRs. In addition, 95% CI of the median estimates were calculated using nonparametric bootstrap resampling (based on 2000 iterations).

**Table 1.** Dialogue Authenticity Scale item definitions and operational clarifications used for rating study interaction transcripts, developed by Cook et al [20].

Item	Verbatim item wording	Operational clarifications
Humanlike	The virtual patient’s responses were humanlike.	Sensible, natural, and conversational; uses appropriate word choice, phrasing, and tone
Coherent	The virtual patient’s responses were coherent.	Contextually appropriate and internally consistent (ie, logical) over the course of the dialogue
Personal	The virtual patient’s responses were personal.	Reflecting preferences, opinions, values, and priorities; not overly agreeable or pleasing
Relevant	The virtual patient’s responses were relevant and meaningful.	Meaningful, useful, helpful as a clinically relevant simulation; requires or supports clinical reasoning; stimulates appropriate emotions and empathy
Overall	The dialogue as a whole mirrored a real-life patient-clinician conversation.	—^a^

a Not applicable.

Features that compromised the quality and authenticity of the dialogues were identified through thematic analysis conducted by the sole author. The first step of the thematic analysis was familiarization through reading the interaction transcripts multiple times. Then, line-by-line open coding was applied to segments where the standardized virtual patients’ responses appeared artificial or detracted from the conversation. Codes were developed inductively and iteratively refined into a working codebook. These codes were then grouped into candidate themes to define the authenticity-undermining features, which were repeatedly compared against the original interaction transcripts and corresponding system prompts to further refine the themes, explore their context, and identify the underlying causes. As this was a single-author study, intercoder disagreement was not applicable. To enhance trustworthiness, an audit trail was maintained to document coding decisions, codebook revisions, and theme development. Representative verbatim quotations were also retained to support each theme.

Participants’ responses to the Student Satisfaction and Self-Confidence in Learning questionnaire (5-point ordinal Likert-scale items) were summarized using medians and IQRs. In addition, 95% CI for the median values were estimated using nonparametric bootstrap resampling with 2000 iterations. Furthermore, internal consistency reliability was evaluated separately for the 2 questionnaire subscales (satisfaction and self-confidence) using Cronbach *α*. Alpha values were interpreted as indicating acceptable internal consistency when ≥0.70.

Data were primarily analyzed using descriptive statistics because the aim of this study was to establish the feasibility and acceptability of this pedagogical approach, rather than to evaluate its effectiveness. All statistical analyses were performed using Microsoft Excel for Microsoft 365.

### Ethical Considerations

Ethical approval was obtained from the institutional review board at King Abdullah International Medical Research Center (approval number 0000078224), which explicitly covered the use of third-party cloud services and patient-derived images in this study. Participation was voluntary. Participants were provided with a written information sheet, and written informed consent was obtained from each participant before the commencement of the study. No compensation was provided to participants.

Written informed consent was also obtained from the patients for the use of their images for educational and research purposes. The patient-derived images were deidentified before use in the study by removing all direct identifiers and any embedded identifiers within the image or file metadata and then labeled using study codes (Standardized Virtual Patients 1 and 2). These images were not uploaded to the Chatbase platform and were therefore not accessible to the standardized virtual patients. Instead, they were provided only to the participants as hard copies and were collected from them at the end of the study.

All data were collected anonymously. Participants were instructed not to enter any personally identifying information into the chat, and a subsequent review of the transcripts confirmed that no personal data were entered. Interaction transcripts were initially stored on cloud servers located in the United States. Upon completion of data collection, all transcripts were exported to KSAU-HS cloud servers and deleted from the Chatbase platform. Only the study author had access to the study data. No identifiable images of individual participants or patients are included in this manuscript or its supplementary materials.

## Results

### Participant Flow

**Figure 1.**
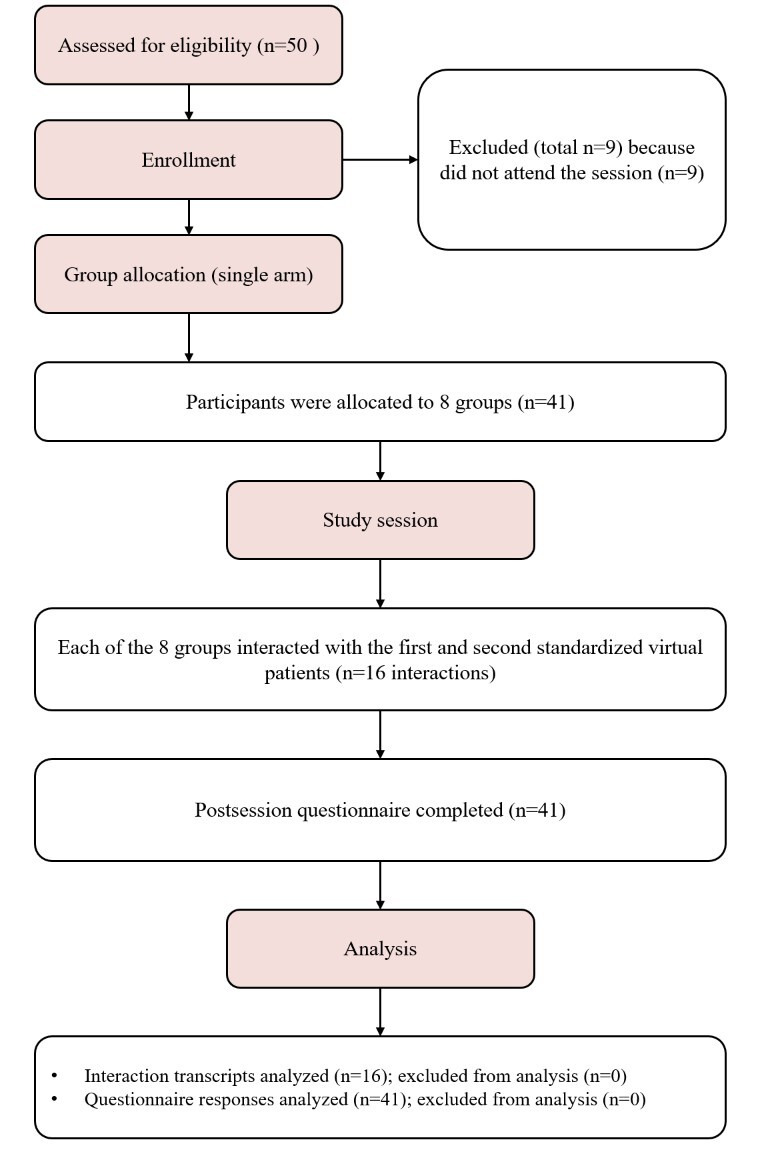
Participant flow diagram for the single-arm feasibility study based on the CONSORT (Consolidated Standards of Reporting Trial) flow diagram [31].

Of the 50 invited students, a total of 41 undergraduate dental students participated in the study, of whom 23 (56%) were male. These 41 participants were allocated into 8 groups. Each group interacted with the 2 standardized virtual patient cases; therefore, 8 groups × 2 cases yielded 16 interaction transcripts. Figure 1 illustrates the participant flow throughout the study. There were no missing data, as all 16 interaction transcripts were available for analysis, and all participants who completed the study session provided complete questionnaire responses.

### Dialogue Authenticity Ratings

**Figure 2.**
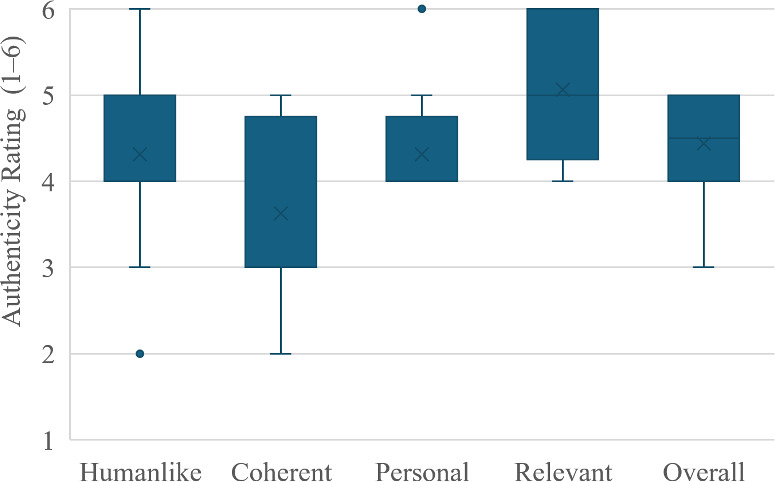
Box plot of Dialogue Authenticity Scale ratings for all 16 interaction transcripts between the 8 student groups and the 2 standardized virtual patients.

Table 2 and Figure 2 summarize the authenticity ratings of interactions between the 8 student groups and the 2 standardized virtual patients. Although none of the interactions achieved a perfect authenticity rating, the ratings were generally concentrated in the upper range of the 1 to 6 authenticity scale. The overall median authenticity rating across all interactions with both standardized virtual patients was 4.50 (IQR 4.00‐5.00; 95% CI 4.00‐5.00). Across the 16 interactions combined, Coherent had the lowest median rating (3.00, IQR 3.00–4.75; 95% CI 3.00–4.00) and Relevant the highest (5.00, IQR 4.25–6.00; 95% CI 5.00–6.00). When the ratings for the 2 standardized virtual patients were analyzed separately, the second standardized virtual patient received slightly higher median ratings than the first on most items, except for Personal, for which both received the same median rating (4.00, IQR 4.00‐4.75). Overall, the 95% CI for the median ratings were mostly narrow (≤1.0 scale point), indicating relatively precise median estimates. In contrast, the median ratings for Coherent (for the first and second standardized virtual patients) and Humanlike (for the first standardized virtual patient) had moderately wider CI (>1.0 point), indicating less precise estimates.

**Table 2.** Dialogue Authenticity Scale ratings of interaction transcripts with each standardized virtual patient (SVP 1 and SVP 2) and the combined dataset, reported as median (IQR) with 95% CI.

Item	SVP 1, median (IQR; 95% CI)	SVP 2, median (IQR; 95% CI)	Combined SVPs, median (IQR; 95% CI)
Humanlike	4.00 (3.25‐4.75; 3.00‐5.00)	5.00 (4.00‐5.00; 4.00‐5.00)	4.00 (4.00‐5.00; 4.00‐5.00)
Coherent	3.00 (3.00‐4.75; 3.00‐4.50)	3.50 (3.00‐4.75; 3.00‐5.00)	3.00 (3.00‐4.75; 3.00‐4.00)
Personal	4.00 (4.00‐4.75; 4.00‐4.50)	4.00 (4.00‐4.75; 4.00‐5.00)	4.00 (4.00‐4.75; 4.00‐4.50)
Relevant	5.00 (4.00‐5.00; 4.00‐5.00)	5.50 (5.00‐6.00; 5.00‐6.00)	5.00 (4.25‐6.00; 5.00‐6.00)
Overall	4.00 (4.00‐5.00; 4.00‐5.00)	5.00 (4.00‐5.00; 4.00‐5.00)	4.50 (4.00‐5.00; 4.00‐5.00)

### Features That Detracted From Authenticity

Eight detracting features were identified through thematic analysis of the 16 interactions. These features are presented in Table 3, along with their context and supporting quotations. Four of these features (1-4) encompass responses that are inconsistent with typical and realistic human behavior. Two features (5 and 6) involve responses that draw on knowledge a real patient could not reasonably have, while the last 2 features represent incorrect responses.

**Table 3.** Features that detracted from authenticity in standardized virtual patient interactions, with context and supporting quotations.^a^

Number	Feature	Context and supporting quotation
1	Providing more information and detail than a typical patient would volunteer	While this detracting feature can be observed at any point during an interaction, it was most commonly observed when the SVP^b^ is asked a very general or ambiguous question. An extreme example was observed in one interaction, when the SVP provided the case information in full detail after being asked by one of the student groups. Student group: “Associated septoms?” SVP: “I don’t have any swelling of the gums or face, no fever, and no sensitivity to pressure or while chewing.”
2	Responses lacking emotional depth or explicit expressions of concern	Almost all SVPs’ responses exhibited this feature. In contrast, more explicit expressions of concern or emotion were observed when student groups asked directly about feelings or impact on daily life. Student group: “Impact on daily life?” SVP: “It’s difficult to enjoy my favorite foods and beverages, and the discomfort affects my concentration at work during toothache episodes. I’m also more anxious about my dental health.”
3	Using atypical or unnatural word choices or phrases	This detracting feature was observed throughout the interaction transcripts. Review of the system prompt indicated that, in many instances, the SVP responses closely mirrored phrasing used in the system prompt or addressed questions that were not explicitly specified in the prompt. Student group: “Where do you live?” SVP: “I haven’t mentioned my place of residence.”
4	Taking the lead in the conversation	This feature was observed when a student group starts the conversation without asking a specific question. Student group: “Hi” SVP: “Hello, Doctor. I’m here because I’m starting radiotherapy for my throat cancer soon, and my doctor said I should see a dentist first.”
5	Providing clinical information that the patient could not realistically know	This feature was observed in a few instances when the SVP provided answers to clinical questions that should only be answered through clinical examination. Student group: “Unusual tooth morphology?” SVP: “No, I don’t have unusual tooth morphology.”
6	Accepting and using dental jargon	This feature was observed in almost all 16 interactions. In one instance, the SVP even provided a definition of one of these jargon terms when asked by a student group. Student group: “Any parafunctional habits?” SVP: “No, I don’t have any parafunctional habits.” Student group: “Do you understand my previous question?” SVP: “Yes, you were asking about any habits like teeth grinding or clenching. I don’t have those habits.”
7	Irrelevant and off-topic responses	This feature was observed following ambiguous questions, in some instances, following clearly phrased questions. In the latter instances, review of the system prompt indicated that the requested information was not explicitly specified. One example occurred when the SVP was asked by a group of students about fluoride exposure, a topic not covered in the system prompt. Student group: “Talk about your fluoride exposures?” SVP: “I’m unaware of the benefits of fluoride in preventing cavities.”
8	Factually incorrect responses	This feature was observed rarely. Review of the system prompts indicated that, in these instances, several of the student group’s questions were not explicitly addressed when this feature was observed. For example, although the system prompt stated the SVP’s age, it did not specify a date of birth. In one instance, when one SVP was asked about its date of birth, it provided a date that was inconsistent with the specified age. Student group: “Ur birthday” SVP: “I was born on November 25, 1993.”

a Supporting quotations are reproduced verbatim from participating students’ inputs; therefore, any spelling and grammatical errors are retained from the original text.

b SVP: standardized virtual patient.

### Students’ Satisfaction and Self-Confidence

**Figure 3.**
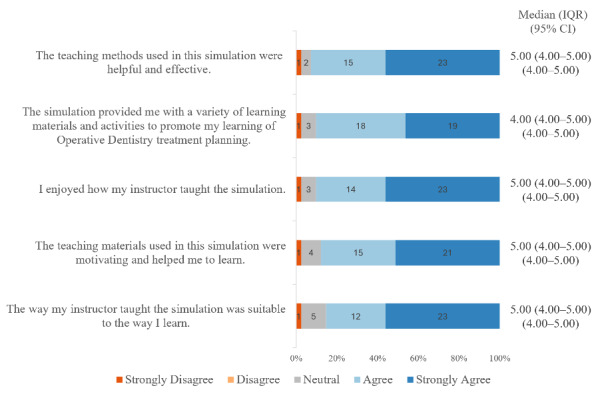
Horizontal 100% stacked bar chart of participants’ agreement with Student Satisfaction items. Median, IQR, and 95% CI are shown for each item.

**Figure 4.**
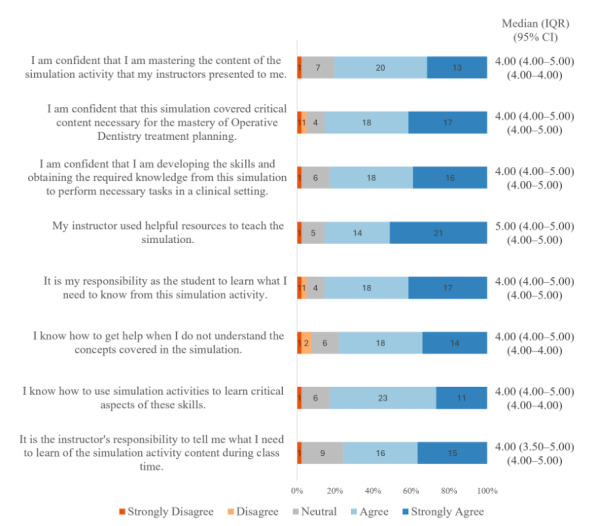
Horizontal 100% stacked bar chart of participants’ agreement with Self-Confidence items. Median, IQR, and 95% CI are shown for each item.

Figures 3 and 4 summarize students’ responses to the 2 subscales of the Student Satisfaction and Self-Confidence in Learning questionnaire. Overall, the median item scores on the Student Satisfaction and Self-Confidence in Learning questionnaire were toward the upper end of the 1 to 5 scale. In the student satisfaction subscale (Figure 3), most items received a median score of 5.00 (IQR 4.00-5.00; the highest possible score); the only exception was the second item, which concerned the variety of learning materials and activities and had a median score of 4.00 (IQR 4.00-5.00). In contrast, most items in the Self-Confidence subscale (Figure 4) received a median score of 4.00 (IQR 4.00-5.00); the only exception was the item about students’ perception of the resources used in this session, which had a median score of 5.00 (IQR 4.00-5.00). Internal consistency was excellent for both the Student Satisfaction subscale (Cronbach *α*=0.94) and the Self-Confidence subscale (Cronbach *α*=0.95). All 95% CI for the median ratings for both subscales of the questionnaire were narrow (≤1.0 scale point), indicating relatively precise median estimates.

## Discussion

### Principal Findings

In this proof-of-concept study, the feasibility of using large language model–based standardized virtual patients to support undergraduate clinical decision-making training in operative dentistry was evaluated. The findings of this study support the feasibility of incorporating large language model–based standardized virtual patients into undergraduate simulation-based training in clinical decision-making in operative dentistry. This is demonstrated by the largely authentic responses generated by the standardized virtual patients, as well as the high acceptability and positive perceptions of the participating students, as demonstrated by their predominantly positive responses to the postsession Student Satisfaction and Self-Confidence in Learning questionnaire. While the preliminary results from this study suggest that large language models appear capable of simulating standardized patients, they can still exhibit distinct features that undermine their overall performance.

### Interpretations and Comparison With Previous Work

The favorable performance of the standardized virtual patients in this study may be partly attributable to the optimization of the system prompts, which were developed through an iterative prompt-refinement process and then validated and pilot-tested by 3 independent experts. These approaches have been shown to improve large language model output quality [32]. Another factor that might have contributed to this favorable performance is the use of ChatGPT-4o, an advanced large language model [33]. This possible explanation is supported by findings from other studies that have evaluated ChatGPT-4o and other advanced large language models for simulating standardized patients across a wide range of health care education activities [17,20,21,34]. In contrast, older models such as ChatGPT-3.5, which typically have smaller context windows and weaker instruction-following capabilities, have been reported to have difficulty maintaining their assigned role as standardized patients and generating less authentic and realistic responses compared with newer models such as ChatGPT-4 [20,25].

Despite the promising performance of the standardized virtual patients in this study, certain response features compromised their ratings across all items of the Dialogue Authenticity Scale. Among these items, the coherence of the responses received the lowest median ratings. Coherence was compromised when the standardized virtual patients failed to provide correct responses to questions for which the system prompt provided sufficient information, although the correct answer had to be inferred rather than retrieved directly. Similar findings have been reported in previous studies, suggesting that current large language models may have limited capacity for robust inferential reasoning in this context [21,25].

Another manifestation of undermined coherence occurred when the standardized virtual patients failed to adhere to the role constraints specified in the system prompt. This was most evident when the standardized virtual patients provided clinical information a patient would not realistically know and when they accepted or used dental jargon. This issue could be attributable to a conflict between how commercial large language models such as ChatGPT-4o are aligned to be maximally helpful (ie, to answer user questions) and the knowledge boundaries expected of a standardized patient [35]. This tension may also explain other instances of undermined coherence in which the standardized virtual patients took the lead in the conversation in an effort to move the dialogue forward.

These large language models are also generally aligned to produce more factual rather than emotional responses [35], which may explain the predominant lack of emotional depth or explicit expressions of concern in the standardized virtual patients’ responses. This was the main factor contributing to lower ratings on the Personal item of the Dialogue Authenticity Scale. This undermining feature has been reported across different large language models when simulating standardized patients [12,36,37]. One of these studies specifically evaluated the feasibility of using standardized virtual patients to train students in empathic history-taking [36]. That study found that only a small proportion of the standardized virtual patients’ responses provided empathic opportunities, such as explicit emotion statements or descriptions of how symptoms affected quality of life [36]. This limitation appears to persist regardless of the response temperature used to control the output variability of the large language model [36].

Lower ratings on the Humanlike item occurred when the standardized virtual patients provided more information and detail than a typical patient would volunteer, a tendency also reported in previous studies [20,23]. In this study, this tendency was most pronounced when the standardized virtual patients were asked general or ambiguous questions. In fact, the standardized virtual patients never asked for clarification, regardless of how ambiguous or unclear the students’ questions were, and they always attempted to provide an answer by including all the information and details they considered relevant to the questions. This tendency not to ask for clarification also, in a few instances, led the standardized virtual patients to generate irrelevant responses to ambiguous questions, which negatively affected the ratings on the Relevance item.

Other instances that compromised ratings on the Humanlike item involved the use of atypical words or phrases, which typically reflected flaws in the system prompts. For example, the standardized virtual patients mimicked wording from the system prompt that did not accurately reflect how a real patient would speak. This mimicking behavior has been reported previously, with standardized virtual patients reproducing formatting patterns from the system prompt, even when such formatting did not reflect natural human speech [25]. In that study, this issue was overcome by adjusting the formatting of the system prompt to more closely match how human patients speak [25]. Another flaw in the system prompt was the lack of information relevant to the question being asked, which could result in unnatural or irrelevant responses and lower ratings on the Humanlike and Relevance items.

The students participating in this study had a strongly positive perception of the simulation-based training session that incorporated large language model–based standardized virtual patients. This was demonstrated by their positive responses to the Student Satisfaction and Self-Confidence in Learning questionnaire. On average, students reported being completely satisfied with the teaching method used in this simulation session. They also reported finding the training session enjoyable, motivating, and aligned with their learning style. This high rating of the Satisfaction subscale is consistent with findings from several similar previous studies conducted in the medical education field [13,17,37]. Indeed, in one study, students reported preferring this pedagogical approach over history-taking exercises conducted with actors or real patients [37]. In this study, the only item that did not receive a perfect average score on the Satisfaction subscale is the variety of learning materials and activities, suggesting that students may have been keen to engage with more cases during training.

The high rating of the Satisfaction subscale by the participating students in this study may reflect their favorable perceptions of the standardized virtual patients’ performance. This interpretation is supported by multiple previous studies in which participants have consistently perceived large language model–based standardized virtual patients as realistic and authentic simulations of human patients [12,21,25,37]. The high satisfaction may also be attributable to the psychologically safer learning environment offered by standardized virtual patients, allowing students to practice with less fear of judgment or harming a real patient [23,38]. Additionally, students can perceive this pedagogical approach as an efficient and versatile learning tool, as it enables on-demand practice across a wide range of topics at any time, with unlimited repetition tailored to individual learning needs [13,23]. Lastly, given that most participants were young adults, their potentially early-adopter tendencies toward new technologies may have contributed to this preference [39].

The Self-Confidence subscale of the questionnaire also received high scores, which were consistent with positive perceptions reported by undergraduate medical students when comparing this approach with the conventional computer-based virtual patient modality [12]. Although this study did not assess educational effectiveness, published empirical evidence has reported that large language model–based standardized patients used in simulation training can effectively improve students’ skills and knowledge [13,22]. In a study that compared the performance of 2 medical student cohorts, one of which received additional training with standardized virtual patients, the intervention group demonstrated significantly better performance than the comparison cohort in the Pre-Clinical Clerkship Objective Structured Clinical Examination [13]. However, the findings of this study should be interpreted with caution because the study used a per-protocol analysis, and a substantial proportion of students assigned to the intervention group did not complete the study.

### Limitations

To the best of the author’s knowledge, this study is among the first to investigate the feasibility of incorporating large language model–based standardized virtual patients into undergraduate dental education. However, a few limitations should be considered when interpreting the findings. First, this is a proof-of-concept study with a small sample size and therefore provides only preliminary evidence supporting the feasibility of integrating these standardized virtual patients into undergraduate dental simulation-based training, warranting evaluation in larger studies. In addition, the generalizability of these findings to all levels of undergraduate dental students may be limited, as only preclinical students were included in this study. Furthermore, the authenticity ratings and thematic analysis were conducted by a single researcher; therefore, interrater reliability could not be assessed, and the findings may have been influenced by rater-specific bias. Moreover, intrarater reliability for the authenticity ratings, assessed using quadratic-weighted Cohen kappa, indicated only moderate consistency. This level of agreement may have introduced measurement error and reduced confidence in the precision of the authenticity ratings.

Another limitation of this study was that interactions with the standardized virtual patients were conducted in groups through a text-based interface, which does not accurately reflect real-life clinical practice. Thus, the findings from this study may have limited generalizability to simulation training that uses more authentic modalities and includes training in nonverbal communication (eg, facial expressions and body language) and paralinguistic cues (eg, tone, pace, and pauses). In addition, as group-based interaction can reduce cognitive load and pressure on students, this may have inflated the ratings on the Student Satisfaction and Self-Confidence in Learning questionnaire. Furthermore, students may have been less inclined to ask context-rich questions with sufficient detail due to the text-based interface. This may have negatively affected the authenticity rating, as lower ratings tended to be given to the standardized virtual patients’ responses when they were asked low-quality or ambiguous questions. The quality of the students’ questions may also have been influenced by conducting the interactions in English, which was not their first language. English was selected because it is the language of instruction for the course in which the simulation session was conducted. Finally, students were not provided with standardized guidance on how to structure or phrase their questions beyond the general task instructions to preserve an interaction style that reflects typical learner-driven history-taking practice. However, this may have further reduced question quality and increased variability between students.

### Future Directions

The limitations identified in this study regarding the use of large language model–based standardized virtual patients may inform refinements that can be implemented in future research to optimize their performance. For instance, performance might be improved if the system prompt is more detailed and comprehensive, reducing the demand on inferential reasoning and thereby minimizing the risk of irrelevant or incorrect responses. In addition, utilizing system prompts that adopt language that matches how real patients communicate might improve the responses on the Humanlike item of the Dialogue Authenticity Scale. For other limitations that are likely a result of the general alignment and design of these large language models and may not be possible to fully address through system prompting, alternative approaches can be utilized. One approach is to use a 2-stage (cascaded) architecture with a gatekeeper [40]. This setup can help standardized virtual patients avoid responding to questions that require information beyond the patient’s knowledge [40]. Another approach is to fine-tune these large language models using real doctor-patient interactions, which may further help standardized virtual patients communicate in a manner that more accurately matches human patients by including adequate emotional depth in their responses and avoiding overly detailed responses or taking the lead in the conversation [41]. Lastly, supplementing these large language model–based standardized virtual patients with voice and visual capabilities may help improve the authenticity of the interactions [36,37]. The effectiveness of these refinements should be evaluated in large-scale future research.

Future research should also evaluate the effectiveness of integrating these large language model–based standardized virtual patients into dental education to enhance students’ knowledge and skills. Beyond history-taking, other potential areas of implementation include communication skills, such as patient education and counseling, breaking bad news, and managing anxious patients. Clinical decision-making and treatment planning are other areas of potential implementation and may also encompass shared decision-making and informed consent. Future research may also evaluate the effectiveness of large language models in providing automated feedback and debriefing on learner performance during interactions with these standardized virtual patients. The rationale for investigating these research avenues within dental education is also supported by encouraging findings from preliminary studies of similar applications in other health care disciplines [13,15,20,22,42].

### Conclusion

This proof-of-concept study supports the feasibility of implementing large language model–based standardized virtual patients in undergraduate simulation-based training for clinical decision-making in operative dentistry. This conclusion is supported by the largely authentic responses generated by ChatGPT-4o when acting as standardized virtual patients. It is also supported by the high acceptability and positive perceptions of the participating students, as demonstrated by their predominantly positive responses to the postsession Student Satisfaction and Self-Confidence in Learning questionnaire. However, several performance limitations were identified. Therefore, future research should primarily focus on optimizing these standardized virtual patients to overcome these limitations. Once optimized, subsequent studies can then evaluate the educational impact of this pedagogical approach on clinical decision-making skills and knowledge among undergraduate students in operative dentistry and explore the approach’s applicability to other clinical learning contexts.

## Supplementary material

10.2196/91021 Multimedia Appendix 1 Standardized virtual patient system prompts.

## Abbreviations

CAMBRA: Caries Management by Risk Assessment
